# Endoscopic Diagnosis of Gastrojejunocolic Fistula After Gastroenterostomy With Billroth II

**DOI:** 10.14309/crj.0000000000001915

**Published:** 2025-12-05

**Authors:** Rahul Karna, Thayer Nasereddin, Muhammad Ali Butt, Elie Aoun

**Affiliations:** 1 Division of Gastroenterology, Hepatology and Nutrition, University of Minnesota, MN; 2 Indianapolis Gastroenterology & Hepatology, Indianapolis, IN; 3 Division of Gastroenterology, Hepatology and Nutrition, Allegheny Health Network, Pittsburgh, PA

## CASE REPORT

**Figure 1.**
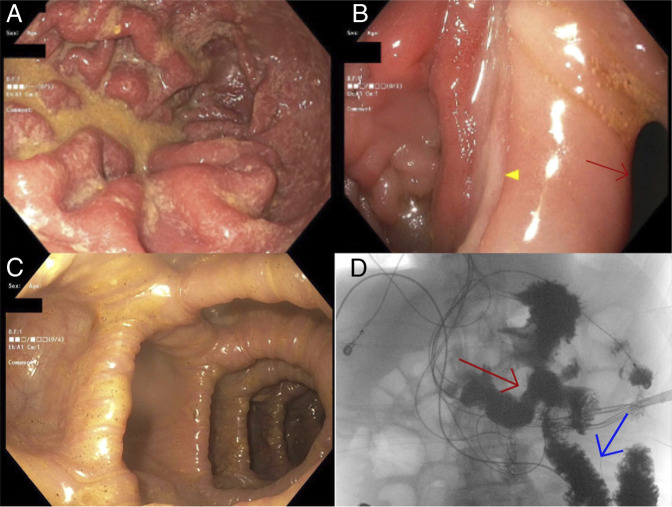
(A) Gastric mucosa with feculent material. (B) Gastroenterostomy with marginal ulcer (yellow arrow); gastrojejunocolic fistula (red arrow). (C) Transverse colon visualized after traversing gastrojejunocolic fistula. (D) Small bowel follow-through with contrast passing preferentially in the jejunum (blue arrow) with retrograde flow into the transverse colon (red arrow).

A 51-year-old man with a history of peptic ulcer disease, complicated by perforation status postpartial gastrectomy with gastrojejunostomy (Billroth II) 6 years prior, presented with chronic diarrhea, intermittent feculent vomiting, severe weight loss, and fatigue. His symptoms had been ongoing for 4 months but progressively worsened over the past month. Laboratory evaluation revealed hypokalemia, hypoalbuminemia, and hypoproteinemia. Initial computed tomography scan with intravenous contrast alone showed no evidence of intestinal obstruction. Metabolic, hormonal, and infectious workup, including Clostridium difficile, was unrevealing. Upper endoscopy showed evidence of feculent liquid in gastric cavity (Figure 1). Anatomy was consistent with previous partial distal antrectomy and gastrojejunostomy, and a clean base marginal ulcer was observed (Figure 1). Efferent jejunal limb was characterized by healthy-appearing mucosa (Figure 1). Interestingly, another tract was identified at the anastomotic site leading into transverse colon, raising suspicion of a gastrojejunocolic fistula, approximately 25 mm in largest cross-sectional diameter (Figure 1). The fistula likely developed secondary to chronic transmural erosion of a marginal ulcer with gradual extension into the adjacent transverse colon, resulting in a mature gastrojejunocolic tract. A subsequent small bowel follow-through demonstrated contrast preferentially passing into the jejunum with retrograde flow into the transverse colon, confirming gastrojejunocolic fistula (Figure 1). The patient was started on total parenteral nutrition due to severe malabsorption and sarcopenia, with plans for surgical fistula takedown once nutritional status was optimized. Gastrojejunocolic fistula is a late complication of gastrojejunostomy and classically presents with diarrhea, feculent vomiting, and weight loss.^1^ Diagnosis is established by demonstrating communication between the gastric or jejunal limb and the transverse colon.^1,2^ Upper gastrointestinal endoscopy, computed tomography, or oral contrast studies can confirm the diagnosis.^2,3^ Management of chronic gastrojejunocolic fistula is primarily surgical, after optimization of nutritional status.^3^ Alternatively, in nonsurgical candidates with acute or subacute, smaller fistulas, endoscopic closure may be considered^4^

## DISCLOSURES

Author contributions: R. Karna: design, drafting, revision. T. Nasereddin: design, draft. MA Butt: draft, revision. E. Aoun: concept, design, obtaining images, supervision, administrative and clinical support. R. Karna is the article guarantor.

Financial disclosure: None to report.

Informed consent was obtained for this case report.
